# Disordered eating & body image of current and former athletes in a pandemic; a convergent mixed methods study - *What can we learn from COVID-19 to support athletes through transitions?*

**DOI:** 10.1186/s40337-021-00427-3

**Published:** 2021-06-24

**Authors:** Georgina Louise Buckley, Linden Elizabeth Hall, Annie-Claude M. Lassemillante, Regina Belski

**Affiliations:** 1 Department of Nursing and Allied Health, Swinburne University of Technology, John St, Hawthorn, Victoria 3122 Australia; 2 Charles Perkins Centre, Inside Out Institute, Sydney University, John Hopkins Drive, Camperdown, New South Wales 2050 Australia; 3 Victorian Institute of Sport, Aughtie Drive, Albert Park, Victoria 3206 Australia; 4 Department of Dietetics, Human Nutrition and Sport, LaTrobe University, Bundoora, Victoria 3083 Australia

**Keywords:** COVID-19, Athlete, Sport, Retired athlete, Eating disorder, Body image

## Abstract

**Background:**

The COVID-19 pandemic has seen worsened mental health as a result of lockdowns, isolation and changes to sociocultural functioning. The postponement of the Tokyo 2020 Olympics is representative of global cancellations of sporting events, reduced facility access and support restrictions that have affected both current and former athlete’s psychological wellbeing. This study aimed to determine whether current (*n* = 93) and former (*n* = 111) athletes experienced worsened body image, relationship with food or eating disorder symptomatology during acute COVID-19 transitions.

**Methods:**

The study was a Convergent Mixed Methods design whereby qualitative content analysis was collected and analysed simultaneously with quantitative cross-sectional data using the EAT-26 and self-report COVID-19 questions. Data were collected from April until May 2020 to capture data pertaining to transitions related to the pandemic and included individuals across 41 different individual and team sports from club to international competition levels.

**Results:**

There was a surge in disordered eating in current and former athletes as a result of the early COVID-19 response. Eating disorders were suggested to occur in 21.1% of participants (18% current athletes *n* = 17, 25% former athletes (*n* = 26). There was a significant difference between males and females (*p* = 0.018, *r* = 0.17), but interestingly no differences between groups from individual vs team sports, type of sporting category (endurance, antigravitational, ball sport, power, technical and aesthetic) or level of competition (club, state, national or international). 34.8% (*n* = 69) self-reported worsened body image and 32.8% (*n* = 65) self-reported a worsened food relationship directly from COVID-19. Qualitative analysis indicated that disordered eating occurred predominantly in the form of body preoccupation, inhibitory food control, fear of body composition changes and binge eating.

**Conclusions:**

This study indicates that transitions in COVID-19 have worsened food-body relationships in current and former athletes and must be treated as an at-risk time for eating disorder development. We suggest that resources are allocated appropriately to assist athletes to foster psychologically positive food and body relationships through COVID-19 transitions. This study makes practice suggestions in supporting athletes to manage control, seek support, adapt and accept change and promote connection and variety through athletic transitions.

**Supplementary Information:**

The online version contains supplementary material available at 10.1186/s40337-021-00427-3.

## Plain English summary

The current study looked to explore how current and former athletes’ disordered eating had been affected in response to the COVID-19 pandemic. We found that disordered eating had worsened in both population groups and was described through increases to food preoccupation, control, binge eating, dietary restriction and fear of body composition change. This study offers suggestions on how to best support current and former athletes through COVID-19 transitions, further relating these findings to athletic transitions such as retirement, injury, pregnancy and illness.

**Supplementary Information:**

The online version contains supplementary material available at 10.1186/s40337-021-00427-3.

## Background

The severe acute respiratory syndrome coronavirus 2 (SARS-CoV-2) pandemic, otherwise known as COVID-19 has caused significant and long-lasting devastation to the mental health of individuals globally [1–5]. Athletes are a particular population group whose identity, careers, socioeconomic circumstances and physical and mental health continues to be impacted by the pandemic [6, 7]. Eating disorders (ED) are complex mental illnesses, characterised by a clinical diagnosis, that are strongly influenced by sociocultural factors [8–11] and as a result of the pandemic have seen increases to the rates of diagnosis and worsened symptomatology of those with pre-existing conditions [12–14]. Disordered eating (DE) is a subclinical state that remains distinguished yet related to EDs and is characterised with less psychological mediation and more transient cognitive and behavioural symptomatology [15, 16].

Individuals can develop worsening DE when their psychosocial circumstances change [9–11, 17–19]. In athletes, DE can develop as a response to control acute transitions such as retirement, injury, coaching changes, illness and is suspected to have occurred through the transitions related to COVID-19 [16, 20–22]. Whilst necessary to control the COVID-19 viral spread, ongoing quarantines, lockdowns, and physical or social isolation have been related to negative psychological affects including that of worsened DE [2, 14, 23–25]. Stress related specifically to the pandemic has explained increases to worsened body image [14, 26]. Individuals who have developed EDs during the pandemic have been further impacted in their access to timely treatment with specialist services experiencing prolonged waitlists, increased referrals and increased help seeking through immediate support resources [20]. Healthcare systems have further seen sub-acute conditions deprioritised contributing to less access for individuals with EDs or DE [27].

In an Australian general population there was increased DE with rises to restrictive dietary practices and binge eating whilst ED populations have seen increases to these symptoms and increases in dysfunctional exercise [28]. Haddad, Zakhour [29] saw that a higher fear of COVID-19 or increased physical activity were associated with a higher dietary restraint whilst fear of COVID-19, higher anxiety and engaging in physical exercise were associated with higher shape and weight concerns. Keel, Gomez [30] described how perceived weight gain or the fear of gaining the “quarantine 15 [pounds]”, rather than actual weight gain, was strongly related to concerns of weight, shape and eating. Castellini, Cassioli [31] reported an increase in objective binge eating and compensatory exercise in those who already had ED diagnostics, whereas no difference in these practices in healthy controls.

Prior to COVID-19, current and former athletes were already seen as at-risk population groups with estimates of EDs occurring in rates three times as often than the general population [16, 32–36]. Considering how much psychosocial factors contribute to DE development, COVID-19 is anticipated to have generated significant changes to athletes food-body relationship especially given the closure of gyms and training facilities, reduced access to healthcare provision including injury rehabilitation and management, limited team access including the central role of teammates and coaches, and the cancellation of competitions [37–41]. The postponement of the 2020 Tokyo Olympics is of particular note for elite athletes as it is an event that individuals commit many years towards whereby the psychosocial preparation towards the event is significant and psychological distress has been anticipated in those this postponement has affected [37, 42–44]. Typically, in moments of stress, athletes have been seen to enlist in additional social support, utilise cohesive training environments and direct attention to their training and competition goals [45, 46]. Many of these typical coping strategies have been affected in one way or another from the pandemic and it is anticipated that for many individuals, their coping strategies will involve symptoms of DE (i.e. dysfunctional exercise, control over food, body preoccupation, dieting, behaviours of compensation, dietary restriction etc.) [21].

Whilst we have little indication of the longitudinal or acute effect that COVID-19 has had on current and former athletes DE state, we can utilise pre-existing literature to hypothesise their responses to times of transition and uncertainty. Former athletes in their acute retirement phase, or current athletes experiencing injury, illness or career interruptions are of particular interest in relation to DE development in times of transition [16]. Previous literature in former athletes has indicated athletes transitioning out of sports experience worsened body image in the acute transition (from 1 to 4.5 months) and individuals turn to controlling their food as a means to cope with other changing facets of their lifestyle [16, 47]. Current athletes experiencing injury, illness, social/relationship issues, illness of family members, burnout and loss of a coach have responded similarly to former athletes and are particularly at risk for DE development at these transitioning times [45, 48]. The context of COVID-19 is of specific interest as it is a transition that places current and former athletes at risk of DE and many of the aforementioned factors are further exacerbated.

At the time of publication there are no studies looking at the state of DE in athletes during COVID-19 and as such we can hypothesise that these at-risk population groups will have experienced worsened DE and body image as an acute affect of the pandemic and the related secondary factors of food insecurity, unemployment, healthcare inaccessibility, social isolation, weight gain fear-mongering, worsened psychological affects and changes to sporting operations. For the purpose of this study we will be interested in exploring how and if DE has developed in current and former athletes as a result of the COVID-19 response and discuss what we can learn from the findings to assist athletic populations in times of transitions. For this convergent mixed methods study we ask the following research question; how have current and former athletes’ acute relationship with food and body, including DE, been affected as a result of a pandemic? The research question aims to be answered in the following two ways: (a) determine whether current and former athletes experienced worsened body image, relationship with food, and DE symptomatology during COVID-19, and (b) explore how current and former athletes’ relationship with food and body has been affected as a result of COVID-19.

## Methods

### Study design and context

This study is a cross sectional convergent mixed methods (CMM) design; where qualitative and quantitative data were collected concurrently via Qualtrics, an online survey program [49]. A specific type of CMM variant will be used to describe the methodology of this study, the questionnaire variant. The questionnaire variant form of CMM is when both open and closed-ended questions are asked in a survey and are used to validate and explain one another [50]. Participants were included in the final data analysis if they (a) were over the age of 18, (b) completed at least the quantitative or qualitative section to completion, and (c) identified as either a current or former athlete. Participants were allowed to participate from any country but were excluded from the data analysis if they only partially completed the quantitative or qualitative section. The data were collected between 29th April and 7th of May 2020. Most countries globally were under similar lockdown restrictions at this time; encouraging citizens to remain at home under unless for highly essential purposes, engage in social distancing and increased hygiene measures. Lockdowns and restricted travel had been enforced since March 2020 with many changes expecting to take months and potentially years to slowly ease out of.

### Procedure and data collection

The study was given institutional ethics approval (Swinburne University Ethics Committee: SHR: 2019/113) and participants consented prior to participation. The quantitative and qualitative data were collected online through convenience sampling; a type of random sampling as it was the most feasible way to recruit individuals given the global lockdowns, the timely nature of capturing athletic transitions and accessibility to social media for participants at the time [51]. Shared social media posts by the primary researcher on Twitter, Instagram and Facebook allowed access to a global recruitment. No procedural differences existed for participants recruited from different countries. The Facebook post was shared 82 times, the Twitter post was retweeted 44 times and the Instagram post reached 1721 individuals.

Participants were asked demographic questions in addition to the Eating Attitudes Test-26 (EAT-26) [52] to indicate DE, 2 quantitative self-report questions to indicate change in body image and food relationship and 4 open ended questions to explain qualitative changes in DE related to COVID-19. The EAT-26 is a 26-item scale to indicate the presence of ED symptoms. The scale includes 3 subscales that characterise aspects of DE presentation: (1) Dieting, is defined as the avoidance of fattening foods and a preoccupation with body change, (2) Bulimia and food preoccupation, is defined as obsessive thoughts about food and bulimia nervosa related behaviours, (3) Oral control, is defined as the self-control related with eating in order to control the body [52]. The EAT-26 criterion validity suggests that a score greater than 20 is indicative of an ED (discriminant function: 82.6%) [52]. The internal consistency for the scale is 0.9 in the general population [52] and has ranged between 0.51–0.96 in studies involving athlete populations [53]. In this study population (*n* = 204) the alpha was established to be 0.92.

Two self-reported questions were asked with corresponding qualitative open-ended responses that included; (1) *How has your body image changed since COVID-19?* (2) *How has your relationship with food changed since COVID-19?* A 5-point Likert scale categorised responses (much worse, somewhat worse, about the same, somewhat better and much better). Participants were asked to qualitatively *describe why this has been the case* for both questions through open ended responses. Participants were then asked, *As a current or former athlete, has anything made things more challenging for you over this time,* with the option to describe in an open ended format the challenges they may have faced. An additional open-ended response further asked, *is there anything you would like to add related to how COVID-19 has affected you?*

### Data analysis

Quantitative data were analysed using SPSS v.25 [54] with a *p* value < 0.05 considered as a significant result. EAT-26 data were distributed non-parametrically through a Kolmogorov-Smirnov test. Due to non-parametricity, Chi squares, Mann-Whitney U, and Kruskal Wallis H tests were conducted, all assumptions were met for these results to be validated. Kruskal Wallis tests were corrected with a Bonferroni correction (α = 0.05). We have analysed this quantitative data via male and female current/former athlete groups to give greater depth into any sex differences that may exist. Consistently female athletes have demonstrated higher rates of DE compared to male athletes and we are interested in assessing whether COVID-19 as a stimulus has seen differences to previous sex understandings of DE [19, 34, 55–57]. As for former athletes, there have been two studies utilising validated ED scales so we need to understand whether male or former female athletes are at higher risk of DE development [16].

Content analysis was used to analyse the qualitative data to explore the contextual meaning of what was being said [58, 59]. We utilised conventional content analysis that aims to describe a phenomenon and is often used to describe individuals attitudes or thoughts of such a phenomenon, in this instance (a) body image, (b) relationship with food and (c) challenges arising during COVID-19 [58].

Credibility and rigor in qualitative methods are of importance in developing the reproducibility and reliability of results in sports psychology [60, 61]. Two researchers (GB & LH) immersed themselves in the text by reading it multiple times. Investigator triangulation was achieved through independent coding and evaluation of text impressions [62]. Codes were developed for each response category (worse, about the same, better and challenges arisen) and have been presented as such in the results. An inter-rate reliability consensus of 87.5% of codes occurred. For any discrepancies, codes were discussed and merged to encompass both of the researchers’ interpretations. Further credibility was established through peer-debriefing between GB & LH’s thoughts and impressions of the text, and additionally through the researchers prolonged engagement in the sporting sector [60]. GB identifies as a woman and is a clinical dietitian and former national level 800 m runner, competing in athletics for upwards of 15 years, and LH is both a dietitian and a current elite international female 1500 m runner, having participated in athletics for 17 years. Both researchers reflexively represented the two participant categories of (a) former athlete and (b) current athlete and were able to interpret the qualitative text from the position of their respective lived experiences, enriching the meaning made from the results and understanding through lived experience what these participants were going through. The final codes have been represented through a tree diagram to explain the main reasons as to why current and former athletes have been affected over this time period. Impressions have been discussed and explored alongside recommendations in the discussion [58, 63].

### Participant characteristics

The sample consisted of 204 current (*n* = 93) and former (*n* = 111) athletes aged between 18 and 63 years (M = 27.0, SD = 8.1). This consisted of 14.2% males (*n* = 29) and 85.8% females (*n* = 175). Athletic status was used to categorise current and former athletes with 78 current female athletes, 15 current male athletes, 97 former female athletes and 14 former male athletes. Amongst the former athletes; 4.5% (*n* = 9) retired in the last year, 10.3% (*n* = 21) 1–2 years ago, 16.7% (*n* = 34) 2–5 years ago, 16.2% (*n* = 33) 5–10 years ago and 6.9% (*n* = 14) retired over 10 years ago. Further demographic data is represented in Table 1.

**Table 1:** Additional Demographic Information

Education	Bachelor, Masters or Doctorate Qualification	79.5% (*n* = 162)		
**Employment**	Full time employment	47.5% (*n* = 97)		
**Nationality**	Australia	60.8% (*n* = 124)	Canada	1.5% (*n* = 3)
	USA	26.5% (*n* = 54)	UK	1.5% (*n* = 3)
	New Zealand	4.4% (*n* = 9)	Other	3.4% (*n* = 7)^a^
	Ireland	2% (*n* = 4)		
**Sport**	Track & Field	37.7% (*n* = 77)	Gymnastics	2.5% (*n* = 5)
	Basketball	5.4% (*n* = 11)	Fencing	2.5% (*n* = 5)
	Triathlon	4.9% (*n* = 10)	Ultra/Trail Running	2.5% (*n* = 5)
	Netball	4.4% (*n* = 9)	Volleyball	2.0% (*n* = 4)
	Rowing	3.9% (*n* = 8)	Australian Football	2.0% (*n* = 4)
	Tennis	3.4% (*n* = 7)	Soccer	2.0% (*n* = 4)
	Swimming	2.9% (*n* = 6)	Other	20.6% (*n* = 42)^b^
	Field Hockey	2.9% (*n* = 6)		
**Sporting Categories**	Endurance	42.6% (*n* = 87)	Weight Class	5.4% (*n* = 11)
	Ball Sports	27.9% (*n* = 57)	Technical	3.9% (*n* = 8)
	Power	11.3% (*n* = 23)	Antigravitational	1.5% (*n* = 3)
	Aesthetic	6.9% (*n* = 14)	Other	0.5% (*n* = 1)
**Team vs Individual Sport**	Team	32.8% (*n* = 67)	Individual	67.2% (*n* = 137)
**Highest Competition Level**	Club	17.2% (*n* = 35)	National	38.2% (*n* = 78)
	State	15.7% (*n* = 32)	International	28.9% (*n* = 59)
**Previous Eating Disorder (*n*** **= 67)**	Anorexia Nervosa	14.2% (*n* = 29)	Binge Eating Disorder	3.4% (*n* = 7)
	Bulimia Nervosa	5.4% (*n* = 11)	Other	5.4% (*n* = 11)
	Orthorexia Nervosa	4.4% (*n* = 9)		
**Current Eating Disorder**	No	89% (*n* = 182)	Yes	10.7% (*n* = 22)^c^

^a^Other nationalities included: Argentina (*n* = 1); Hong Kong (*n* = 1); Israel (*n* = 1); Mexico (*n* = 1); Netherlands (*n* = 1); Poland (*n* = 1); South Africa (*n* = 1)

^b^Other sports included: Softball (*n* = 3); Ballet (*n* = 3); Cycling (*n* = 3); Equestrian (*n* = 2); Jump rope (*n* = 2); Kayaking (*n* = 2); Lacrosse (*n* = 2); Muay Thai (*n* = 2); Rock Climbing (*n* = 2); Rugby (*n* = 2); Synchronised Swimming (*n* = 2); Taekwondo (*n* = 2); Weightlifting (*n* = 1); American Football (*n* = 1); Bobsleigh (*n* = 1); Bodybuilding (*n* = 1); Jiu Jitsu (*n* = 1); Goalball (*n* = 1); Ice Hockey (*n* = 1); Irish Dancing (*n* = 1); Kick Boxing (*n* = 1); Boxing (*n* = 1); Powerlifting (*n* = 1); Roller Skating (*n* = 1); Surf Lifesaving (*n* = 1); Touch Football (*n* = 1); Water Polo (*n* = 1)

^c^Current female athletes (*n* = 10), former female athletes (*n* = 12), current/former male athletes (*n* = 0)

### Terminology and definitions

#### Content analysis

Content analysis is the process of coding and categorising text into themes based on their content rather than their thematic interpretations [58].

#### Convergent mixed methods (CMM)

A type of mixed methods project design that combines both qualitative and quantitative data to elucidate new knowledge through interpretations, offering greater insights into a research question than quantitative or qualitative methods alone [50].

#### Current or former athlete

A current or former ‘athlete’ in the instance of this project is somewhere who self identifies as an individual who either currently or formerly participated in sport to a vary degree of competition level and engages with an athletic identity. Athletic identity is the degree to which an individual identifies as an athlete or with an athletic role [64]. Studies engaging athletes often focus on elite athletes and as such, the perspectives of many who strongly identify as athletes across a range of competition levels are often excluded. Current and former athletes from all range of sports were encouraged, including dancers which are often ambiguously categorised.

## Results

### Convergent mixed methods results

**Fig. 1:**
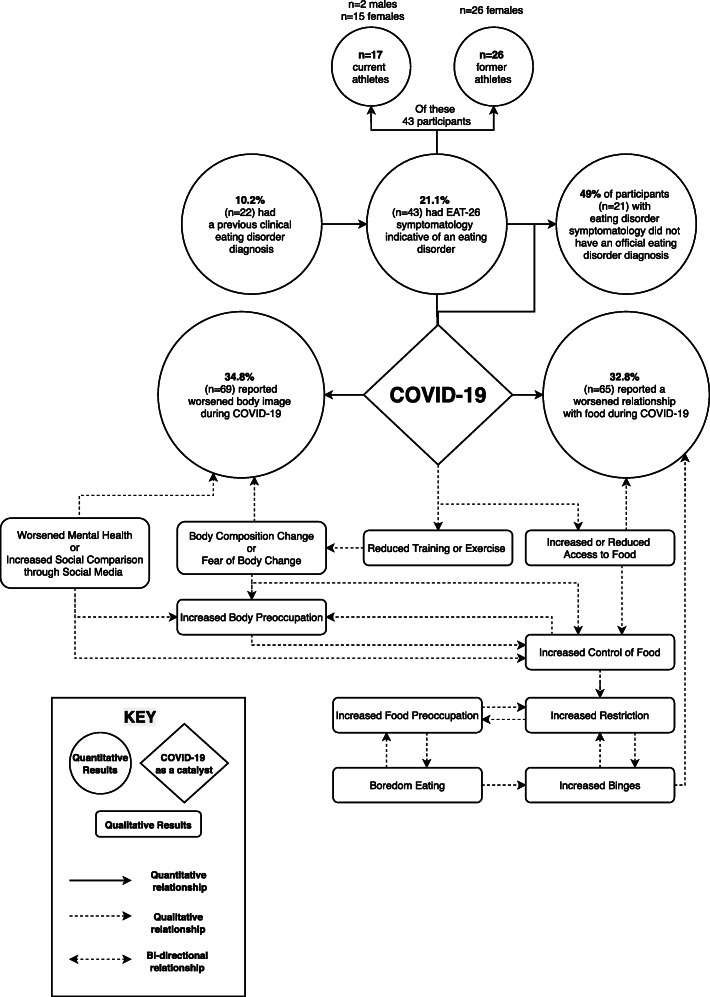
Convergent Mixed Methods Results Diagram – The Convergent Relationship of how COVID-19 has Perceptively Affected Current and Former Athletes’ Relationship with Food and Body Image

Both qualitative and quantitative results have been combined to provide insight into the interrelationship of how COVID-19 has affected current and former athletes’ relationship with food and their body (Fig. 1).

**Fig. 2:**
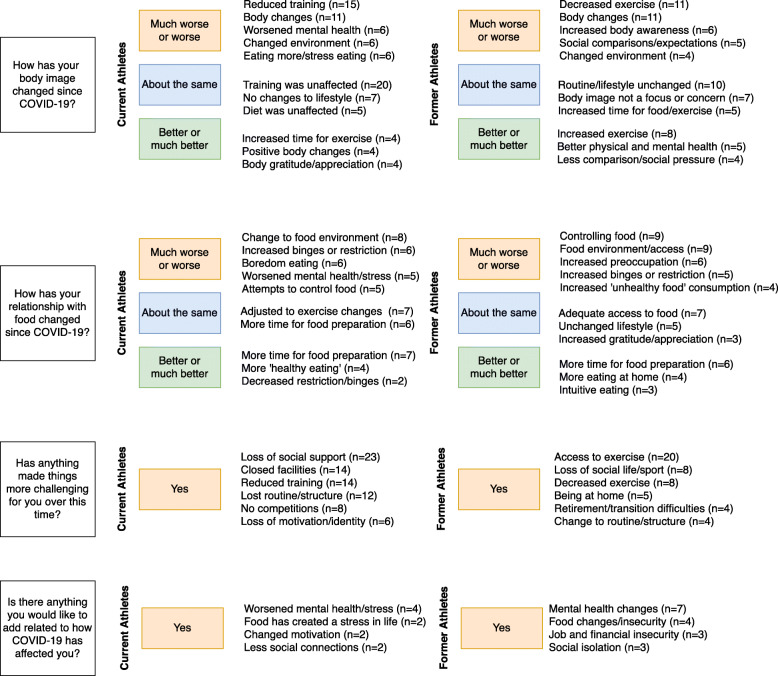
Coding Tree – Qualitative Content Analysis Results Summarised by Corresponding Closed Ended Question and Related Response

Current and former athletes found that changes to their exercise and training were the most significant factor in affecting both their perception of their body and their relationship with food over this time. Reduced training or exercise meant energy balance shifts with subsequent food changes. This was described to result in increased binges, restriction, guilt, shame or increased/decreased control over food. Many described body composition changes that created negative affect towards their body shape and size. Even in the absence of body changes, the fear of anticipatory body changes was enough to indicate a worsened body image. The coding tree summarises the main influences that affected changes to body image and relationship with food in Fig. 2. Given that the main aim of the study is related to DE and negative affect of the food-body relationship, qualitative results related to improved or maintained food-body relationship can be access in the supplementary materials.

#### Worsened body image

A worsened body image was often conflated with body changes, including weight gain or muscle loss, due to exercise and energy balance changes. For many current and former athletes, the ability to exercise was intricately related to the way they perceived their body. Less exercise, training or competitions ensured that participants were fearful of weight gain. It was noted that for current athletes, loss of muscle mass in addition to fat gain was a further stressor as one current athlete described, *“I’m worried that I’m losing fitness, and the more I lose fitness and strength, the more my body changes…I’m feeling sad about being bigger than others...”* One current weight class athlete reported that their preoccupation with energy and calories had increased with the fear of their body composition changing, *“I usually train 4 hrs a day and never have to think about weight or calories I burn unless I’m cutting weight for a competition. I am usually very lean when training and working full time. I exercise every day now, but my energy expenditure is way lower and I love food. I eat well and have a little treat every night and I’m fit, but my body has changed and I’m not as lean as usual, which is a little uncomfortable.”*

A current athlete with an ED described how their symptomatology was worse at the start of isolation, *“My eating disorder voice has gotten significantly louder. However, it was a lot worse at the beginning of our stay at home order (March 13*^*th*^*) than now.”* For others, a changing environment and being at home created body preoccupation and greater comparison to others through social media, as one current athlete reported, *“I’ve been way more conscious of how I dislike some features and way more obsessive, especially with how many posts are on social media.”* For a former athlete, video programs such as Zoom, created additional body dissatisfaction and stress, *“I hate attending Zoom meetings because I compare myself to others on the screen.”* A former athlete noticed body changes and was trying to accept these changes but finding it hard to find clothes to fit their body, “*I’ve gained weight and I have very few clothes left that fit comfortably. Online shopping is hard when you don’t know your new size and you can’t picture clothes that look good on skinny models looking good on me. Gaining a significant amount of weight has challenged who I feel I am and that itself makes me feel guilty.”*

#### Worsened relationship with food

A worsened relationship with food was often attributed to a perceived lack of control or loss of control. Control was commonly used to describe how individual’s relationship with food had changed. It was described to be a coping strategy in addition to something that participants felt they had lost. In an uncertain and unprecedented time, both current and former athletes described attempting to control food to provide a sense of agency over their life, as one former athlete described, “*In this chaotic time I have found food to be a pillar of control - so I feel as if I am exerting greater control over aspects of my diet which is getting somewhat stricter than normal.”* For a current athlete, they felt that their eating had become out of their control, leading to a complex psychological affect of guilt, shame and stress, *“I feel now that I have less willpower to control what I eat. This means I am eating more rubbish and feeling worse about it afterwards. Before the pandemic I used more energy worrying about what I ate, but at least some of it was productive energy, i.e. I would do meal planning etc. Now my mental health has diminished I have less energy to do that so I am eating more junk, and feeling the effects in my body (both real physical effects and worry about gaining weight etc) but feeling pretty powerless to do anything about it. The lack of ability to exercise and play my sport worsens these feelings.”*

Others reported that their living situations had changed or that they were not coping with either (a) an increase to food access at home, or (b) a decrease to food access in their communities. A former athlete described how this had affected their relationship with food, *“I have a lot of anxiety around food and am out of my routine. I have to shop for a couple of weeks at a time instead of getting fresh veg every few days. I can’t have meals with friends so I sometimes just can’t be bothered dealing with the anxiety of food just for myself.”* A current athlete described moving back home had reduced their food options, *“…coming home from college and not having the freedom of my own food choices.”* Whilst a former athlete described how they found the increased access to food at home stressful, *“I am living back home with my parents and have access to a wider variety of food, including ‘bad’ foods. I feel like I lack the control to be in this environment.”*

#### Additional COVID-19 challenges

Current and athletes differed in their reliance on peers, family and friends. For current athletes it was the loss of support that these people were able to give to them that made COVID-19 more challenging whereas with former athletes it was the loss of socialising through sport and social events that felt challenging. The change in exercise/training/competitions was of the most significance for both current and former athletes. A current athlete described how changes to their exercise has highlighted their motivation behind their sport, *“I am not able to train all the time. Which is something I use to make sure I don’t gain weight.”* Former athletes described that exercise was previously used a tool to assist their mental health. As one participant described, *“I can’t access a pool to train. I don’t compete anymore but still feel most safe in the water and deal with my anxiety by swimming, it’s made it so much harder not to be able to swim.”*

For another current athlete, this time was made more challenging as it gave additional time to reflect on some of the more nuanced challenges they are facing in their sport, *“I have quite limited access to training resources at the moment. I already felt like a bit of an impostor because I go to big international competitions, but I represent a country which is very weak in my sport, and I’m an amateur who works full time, and I can’t train as much or to the same level as professional athletes from other countries and this is reflected in my body, my sports ability, and my results. I already felt kind of unworthy of going to these competitions, like I’m not a real athlete (I thought twice about even doing this survey). Now I can see Instagram stories etc both from people I know personally representing other countries and people I only know by reputation and see how much harder they are training than I am, it worsens those feelings of inadequacy.”* Another current athlete reflected on how the postponement of the Olympics has increased the financial pressure they found themselves under, *“[This] has extended the amount of time I have to financially support myself before the Olympics, while training at a high level. Have had to seek further employment to get myself through an extra year.”*

Other athletes described that their reliance on social media in the absence of physical competition had increased. Sites such as Strava were used to compare training distance and speed as a way to increase pressure and social comparison, *“Comparison to others exercise has increased with more time to look on Strava”* in addition to, *“It seems like everyone is working out more. Seeing their workout summaries on Strava makes me feel guilty about how I look and the shape I’m in.”* For others, sport without physical competition was no longer worth the time and energy, “*[It’s challenging] finding ways to be active without being super ‘competitive.’ For me, if I’m not ‘training’ hard it’s a waste of time.”*

During the pandemic, some participants described how they were finding it challenging to transition or retire from sport, whilst others described the difference between this and other times of transition such as injuries. A current athlete described, *“COVID-19 has affected my general mental health quite a lot through isolation and anxiety. I think this is having as much of an effect on my fitness and relationship with my body as anything else. It’s different from an injury because at least with an injury you can train the rest of your body, and other people know how to help. Everyone is going through this at the same time so there isn’t a stable support to lean on. I feel like everyone else is staying on top of things and I am not.”* For one former athlete, they reflected on how their previous sporting transitions has given them a level of resilience to cope with unprecedented times such as this, *“My sporting resilience in the face of uncertainty has actually done me a world of good. Sitting with the unknown of selection has taught me to sit with uncertainty and control why [sic: what] I can control - and remove my focus from the rest.”*

#### Quantitative descriptive statistics & EAT-26 reliability

Descriptive data on continuous variables including; age, height, weight, total EAT-26 scores and subscales are described in Table 2. with corresponding internal consistency reliability statistics (Cronbach’s alpha). The reliability of the EAT-26 total score for the total sample population (*n* = 204) was 0.92 and 0.83 for para-athletes (*n* = 4). Values above 0.7 are considered acceptable [65].

**Table 2:** Descriptive Statistics for Sex and Athletic Status with EAT-26 Total Score and Subscale Scores

	Current Athletes Females (*n* = 78)		Former Athletes Females (*n* = 97)		Current Athletes Males (*n* = 15)		Former Athletes Males (*n* = 14)	
	M (SD)	*alpha*	M (SD)	*alpha*	M (SD)	*alpha*	M (SD)	*alpha*
Age	25.0 (8.5) ^a^		27.0 (7.4) ^a^		26.0 (11.2)		29.5 (6.5)	
Height	166.5 (7.3) ^b^		169.0 (7.5)^c^		176.0 (8.8) ^b^		181.0 (7.5) ^c^	
Weight	61.0 (9.1) ^a,b^		65.0 (12.5) ^a,c^		68.0 (10.2) ^b,d^		83.0 (11.1) ^c,d^	
EAT-26 Total Score	7.5 (13.4) ^b^	*0.93*	6.0 (11.8)	*0.91*	2.0 (7.5) ^b^	*0.88*	3.5 (3.0)	*0.45*
Dieting Subscale	5.0 (8.8) ^b^	*0.92*	4.0 (7.6)	*0.88*	1.0 (7.5) ^b^	*0.78*	2.0 (3.1)	*0.75*
Bulimia Subscale	1.0 (3.0)	*0.70*	0.0 (3.1) ^c^	*0.81*	0.0 (2.3)	*0.86*	0.0 (0.6) ^c^	*0.45*
Oral Control Subscale	1.0 (3.4)	*0.76*	1.0 (2.5)	*0.58*	2.0 (1.1)	*	2.0 (1.1)	*

*Violates reliability model assumptions (inadequate sample size)

^a^Significant difference between females

^b^Significant difference between current athletes

^c^Significant difference between former athletes

^d^Significant difference between males

Non-parametric Independent Samples Mann Whitney U tests were conducted between groups, significance was indicated by *p* < 0.05. There was a significant difference between current male and female athletes in the total EAT-26 score (Males: M = 2.0, *n* = 15; Females: M = 7.5, *n* = 78), U = 781, z = 2.06, *p* = 0.04, *r* = 0.21; and in the dieting subscale (Males: M = 1.0, *n* = 15; Females: M = 5.0, *n* = 78), U = 850, z = 2.78, *p* = 0.005, *r* = 0.29.

21.1% of participants had EAT-26 scores suggestive of an ED, there was a significant difference between males and females (males: M = 3.0, *n* = 29; females: M = 6.0, *n* = 198), U = 3234, z = 2.37, *p* = 0.018, *r* = 0.17, but no significant difference between groups of athletic status, individual vs team sports, type of sport or level of competition (Table 3). The difference between males and females was only significant through the dieting subscale scores (males: 1.0, *n* = 29; females: M = 5.0, *n* = 175), U = 3508, z = 3.32, *p* = 0.001, *r* = 0.23

**Table 3:** Descriptive Statistics of EAT-26 Scores Indicative of an Eating Disorder

**Athletic Status (*n*** **= 204)**	18% current athletes (*n* = 17)	25% former athletes (*n* = 26)
**Individual vs Team Sport (*n*** **= 204)**	19% individual athletes (*n* = 26)	25% team sport athletes (*n* = 17)
**Type of Sport (*n*** **= 204)**	16% endurance athletes (*n* = 14) 19% ball sport athletes (*n* = 11) 36% aesthetic athletes (*n* = 4)	0% antigravitational athletes (*n* = 0) 14% power athletes (*n* = 8) 25% technical athletes (*n* = 2)
**Highest Level of Competition (*n*** **= 204)**	26% club level athletes (*n* = 9) 26% national level athletes (*n* = 20)	16% state level athletes (*n* = 5) 15% international athletes (*n* = 9)

#### COVID-19 responses & EAT-26

A series of categorical COVID-19 questions were asked regarding relationship with food, body image and challenges over this time. The results are presented in Table 4 and indicate the percentages of respondents for each category (worse/same/better; yes/no). EAT-26 total score was compared to these response groups and significant differences assessed. Kruskal-Wallis H Tests were conducted to assess non-parametric one-way between groups analysis of variance between COVID-19 questions 1 and 2, EAT-26 scores and athletic categories. Mann-Whitney U Tests were conducted for question 3, as it only had 2 categorical response options. Bonferroni adjustments were applied when comparing multiple groups.

**Table 4:** COVID-19 Categorical Responses vs. Athletic Status and Sex and Difference Within Response Groups

		All participants (*n* = 198)		Current Athletes Females (*n* = 77)		Former Athletes Females (*n* = 93)		Current Athletes Males (*n* = 14)		Former Athletes Males (*n* = 14)	
		%, n	EAT-26 M (SD)	%, n	EAT-26 M (SD)	%, n	EAT-26 M (SD)	%, n	EAT-26 M (SD)	%, n	EAT-26 M (SD)
1. How has your body image changed since COVID-19?	Worse	34.8 (*n* = 69)	**9.0 (13.4)**^**a**^	42.9 (*n* = 33)	8.0 (14.4)	34.4 (*n* = 32)	**15.0 (12.9)**^**b**^	14.3 (*n* = 2)	15.0 (12.7)	14.3 (*n* = 2)	4.0 (2.8)
	Same	50.5 (*n* = 100)	**5.0 (11.4)**^**a**^	42.9 (*n* = 33)	7.0 (13.6)	52.7 (*n* = 49)	**4.0 (11.1)**^**b**^	71.4 (*n* = 10)	2.0 (6.9)	57.1 (*n* = 8)	3.5 (2.7)
	Better	14.6 (*n* = 29)	5.0 (8.1)	14.3 (*n* = 11)	8.0 (10.7)	12.9 (*n* = 12)	5.0 (5.9)	14.3 (*n* = 2)	2.0 (1.4)	28.6 (*n* = 4)	4.5 (4.0)
2. How has your relationship with food changed since COVID-19?	Worse	32.8 (*n* = 65)	**13.0 (14.5)**^**a**^	46.8 (*n* = 36)	10.5 (14.3)	28.0 (*n* = 26)	**21.0 (14.6)**^**b**^	14.3 (*n* = 2)	13.0 (15.6)	7.1 (*n* = 1)	9.0 (0.0)
	Same	53.0 (*n* = 105)	**5.0 (9.7)**^**a**^	41.6 (*n* = 32)	6.0 (13.3)	55.9 (*n* = 52)	**4.0 (7.9)**^**b**^	71.4 (*n* = 10)	4.0 (6.7)	78.6 (*n* = 11)	3.0 (2.0
	Better	14.1 (*n* = 28)	7.5 (9.1)	11.7 (*n* = 9)	7.0 (11.5)	16.1 (*n* = 15)	8.0 (8.5)	14.3 (*n* = 2)	1.5 (0.7)	14.3 (*n* = 2)	8.5 (3.5)
3. Has anything made things more challenging for you over this time?	Yes	62.6 (*n* = 124)	6.0 (12.7)	76.6 (*n* = 59)	7.0 (13.2)	54.8 (*n* = 51)	**11.0 (13.0)**^**b**^	57.1 (*n* = 8)	2.0 (1.8)	42.9 (*n* = 6)	2.0 (3.7)
	No	37.4 (*n* = 74)	6.0 (10.6)	23.4 (*n* = 18)	9.0 (14.5)	45.2 (*n* = 42)	**4.0 (9.0)**^**b**^	42.9 (*n* = 6)	11.0 (9.8)	57.1 (*n* = 8)	4.5 (2.5)

^a^*p* < 0.05 for within group difference for all participants

^b^*p* < 0.05 for within group difference for former female athletes

#### Body image changes during COVID-19

When looking at all participants, there was a significant difference in EAT-26 total score between those who perceived their body image to have gotten worse as a result of COVID-19 and those who perceived it to have stayed the same, χ^2^ (2,198) =8.01, *p* = 0.018. The group who perceived worsened body image had a higher median EAT-26 score (M = 9.0, SD = 13.4) compared to the group who perceived it stayed the same (M = 5.0, SD = 11.4). This was also the case in former female athletes, χ^2^ (2,81) =7.93, *p* = 0.019 with those experiencing worsened body image having a higher median EAT-26 (M = 15.0, SD = 12.0) compared to the group who perceived their body image stayed the same (M = 4.0, SD = 11.1). There were no significant differences between categories in current female athletes, current male athletes or former male athletes. There was also no significant differences across rows in their EAT-26 scores.

#### Relationship with food changes during COVID-19

When looking at all participants, there was a significant difference in EAT-26 total score between those who perceived their relationship with food to have gotten worse during COVID-19 compared to those who perceived it stayed the same, χ^2^ (2,198) =15.5, *p* = 0.000. The group who perceived their relationship with food to have gotten worse had a higher median EAT-26 score (M = 13.0, SD = 14.5) compared to the group who perceived it stayed the same (M = 5.0, SD = 9.7). This was also seen in former female athletes, χ^2^ (2,81) =15.8, *p* = 0.000, with those experiencing worsened body image having a higher median EAT-26 (M = 21.0, SD = 14.6) compared to the group who perceived body image stayed the same (M = 4.0, SD = 7.9). There were no significant differences between categories in current female athletes, current male athletes or former male athletes. There were no significant differences between categories in current female athletes, current male athletes or former male athletes. There was also no significant differences across rows in their EAT-26 scores.

#### Other results

Former female athletes who perceived things had been made more challenging during COVID-19 had significantly higher EAT-26 scores (M = 11.0, SD = 13.0) than those who did not perceive COVID-19 to be challenging (M = 4.0, SD = 9.0), U = 813, z = − 1.997, *p* = 0.046, *r* = 0.21. No other group had significant differences. There were no significant differences between highest sporting competition (club, state, national, international) and EAT-26 scores (*p* = 0.428); last competition for former athletes (1 month to 30 years +) and EAT-26 scores (*p* = 0.937); sporting categories (endurance, antigravitational, ball sports, power, weight class, aesthetic, technical, other) and EAT-26 scores in current (*p* = 0.149) or former athletes (*p* = 0.519). Additionally, both body image (*p* = 0.673) and relationship with food (*p* = 0.386), current athletes were not significantly different between EAT-26 scores and response categories. There were no significant differences between the major countries analysed in this study (Australian *n* = 121, USA *n* = 51); a chi-square test for independence indicated no difference between changes to relationship with food χ^2^ (2, *n* = 172) = 1.02, *p* = 0.60, Cramer’s v = 0.60, or body image χ^2^ (2, *n* = 172) = 1.82, *p* = 0.40, Cramer’s v = 0.41. A Mann-Whitney U Test revealed no significant differences between the EAT-26 total scores of Australia (Md = 6.0, *n* = 124) and the USA (Md = 5.0, *n* = 54), U = 3156, z = − 0.61, *p* = 0.53, *r* = 0.05.

## Discussion

Our preliminary study has demonstrated that there is evidence of a surge in DE in current and former athletes as a result of the early COVID-19 response. Questions remain about the effect this transition has had on clinical ED rates specifically and the temporal trajectory of such DE states. What this study does indicate is that transitions, such as the COVID-19 pandemic response, for current and former athletes should be treated as an at-risk time for DE development in both males and female. Increased body preoccupation was often linked to increased control of food in a self-perpetuating cycle; leading back to increased body preoccupation and worsened body image. This phenomenon was often related to restrict-binge cycles and a worsened relationship with food. This study has indicated that athletes require specific support in times of transition, such as during the COVID-19 pandemic, due to the close proximity of current and former athletes body composition and physical appearance to their body image and psychological wellbeing.

Former female athletes who experienced worsened DE are further hypothesised to be the most likely group to have developed EDs from the pandemic transitions. What is particularly interesting about those who self-reported their food-relationship had worsened, was that the average score of 21.1 fares higher than the EAT-26 cut-off indicative of ED pathology (EAT score > 20) [52]. This was not seen in current females nor current/former male athletes. Thus, it is hypothesised that female former athletes are the most likely in this population to have developed new ED symptomatology as a result of acute COVID-19 restrictions.

Qualitatively, exerting cognitive control over food was the most commonly indicated factor that female former athletes referred to for developing a worsened food relationship. Control amongst an individual’s food relationship appeared to represent itself paradoxically. Control materialised in a self-perpetuating cycle such that behavioural attempts to control food resulted in reinforced cognitions whereby food ends up preoccupying the individual leading back to further cognitive control, and so on. For former athletes inhibitory control appeared to be described around food behaviours, whereas for current athletes’ control was described related to exercise practices in order to regulate body composition. Similar to Haddad, Zakhour [29] the relationship between exercise and DE or body image concern appeared to be altered through the pandemic. Inhibitory control has a role in maintaining DE and more specifically in athletes it can function to enhance performance focus [66, 67]. It appears that in this study inhibitory control may play a role in modulating stress in such an uncertain transition as the COVID-19 pandemic.

Changes to movement, training, physical activity and energy burning was frequently cited to negatively affect the way that participants related to their body. For former athletes, body image concerns were related to changes in weight or body shape. Whereas for current athletes it was often a fear of altered body composition either through gain of fat or loss of muscle. Body composition changes to current athletes was intricately linked to fitness components; a change in body composition was said to signify a fear of compromised performance ability. Further to this what was most interesting amongst participants was that irrespective of whether the body had actually changed or not, it was the fear of change that substantially indicated a worsened body image. Keel, Gomez [30] further described that in the absence of any weight changes, undergraduate students described their bodies changing significantly. This indicates the significance of the fear of body composition change and its relationship to DE.

Body image is an ambiguous and broad term that can mean a number of things to any one individual [68]. Throughout this content analysis, body image was primarily interpreted by participants as relating to their physical appearance or the physical attributes of one’s body rather than the way they cognitively, emotionally or functionally related to their body [68]. Some did mention how they felt emotionally towards their body, but it still centred the emotional response to changes of physical attributes e.g. I feel sad because I have gained weight. A literature review by Buckley, Hall [16] described how former athletes feel a sense of loss and “body grief” around body changes in times of transitions, particularly athletic retirement. This is suspected to occur when an individual throughout their athletic career develops a sense of athletic identity that encompasses their physical appearance. Thus, changes to their body shape, weight and composition can indicate a loss of that identity and sense of self [16]. This grief, fear, loss, dissatisfaction and negative affect relating to the body is suggested to provide an ideal psychological foundation for ED development [16, 69, 70].

There appears to be a relatively large number of participants that have EAT-26 scores indicative of ED pathology (21.1%, *n* = 43) in this population group, yet 49% of these individuals (*n* = 21) had not received an official ED diagnosis. The body image and food relationship had worsened in > 30% of participants suggests that a large proportion of these individuals have developed worsened ED symptomatology during the acute pandemic response. The results remain inconclusive however, in the absence of a longitudinal study or of quality comparable epidemiological prevalence rates of ED in current and former athletes. Considering that the prevalence of EDs in athletes varies widely from 2 to 42% depending on the type of sport, gender and study rigour [15], it is difficult to understand how 21.1% in this participant group compares to typical rates in athlete populations. There are no comparable quantitative studies relating to former athletes [16], and thus this study is of particular interest as it demonstrates comparable ED rates between current and former athletes, the first study of its kind to demonstrate so. The only prevalence study of former athletes indicated 0.4% of former rowers, wrestlers, boxers and judokas demonstrated ED symptomatology [71].

### What have we learnt about athletic transitions?

As it stands, there are very little studies that explore how athletes respond to transitions in relation to their ED risk. Whilst COVID-19 remains an unprecedented event with long-term implications, we can study its effects to understand how athletes respond to heightened times of uncertainty in relation to their food-body relationship. By studying those who experienced both adaptive and maladaptive responses to their food-body relationship we have been able to draw conclusions that are relevant to navigating future athletic transitions such as retirement, pregnancy, injury and illness. Detailed below is a table describing the findings that have influenced the food-body relationship (Table 5). The first column detail what has been transient and unique to COVID-19, whereas the second column demonstrates the findings from this study that are analogous with athletic transitions.

**Table 5:** The factors that influence the food-body relationship in athletes through COVID-19 and in more generalised athletic transitions

COVID-19 Specific Transitions	Generalised Athletic Transitions
▪ Changes to physical environment ▪ Increased comparison through social media ▪ Changes to food security; including panic buying and fluctuating food access ▪ Boredom eating ▪ Reduced access to physical activity; including access to facilities or time allowed outside due to lockdowns	▪ Increased body preoccupation ▪ Attempts to control food as a surrogate to control life changes ▪ Changes to appetite ▪ Reduced training and physical exercise ▪ Routine and lifestyle changes ▪ Body composition changes; including changes to muscle mass and fat percentage ▪ Altered mental health state; including a change to identify and self-perception ▪ Loss of motivation or direction

**Fig. 3:**
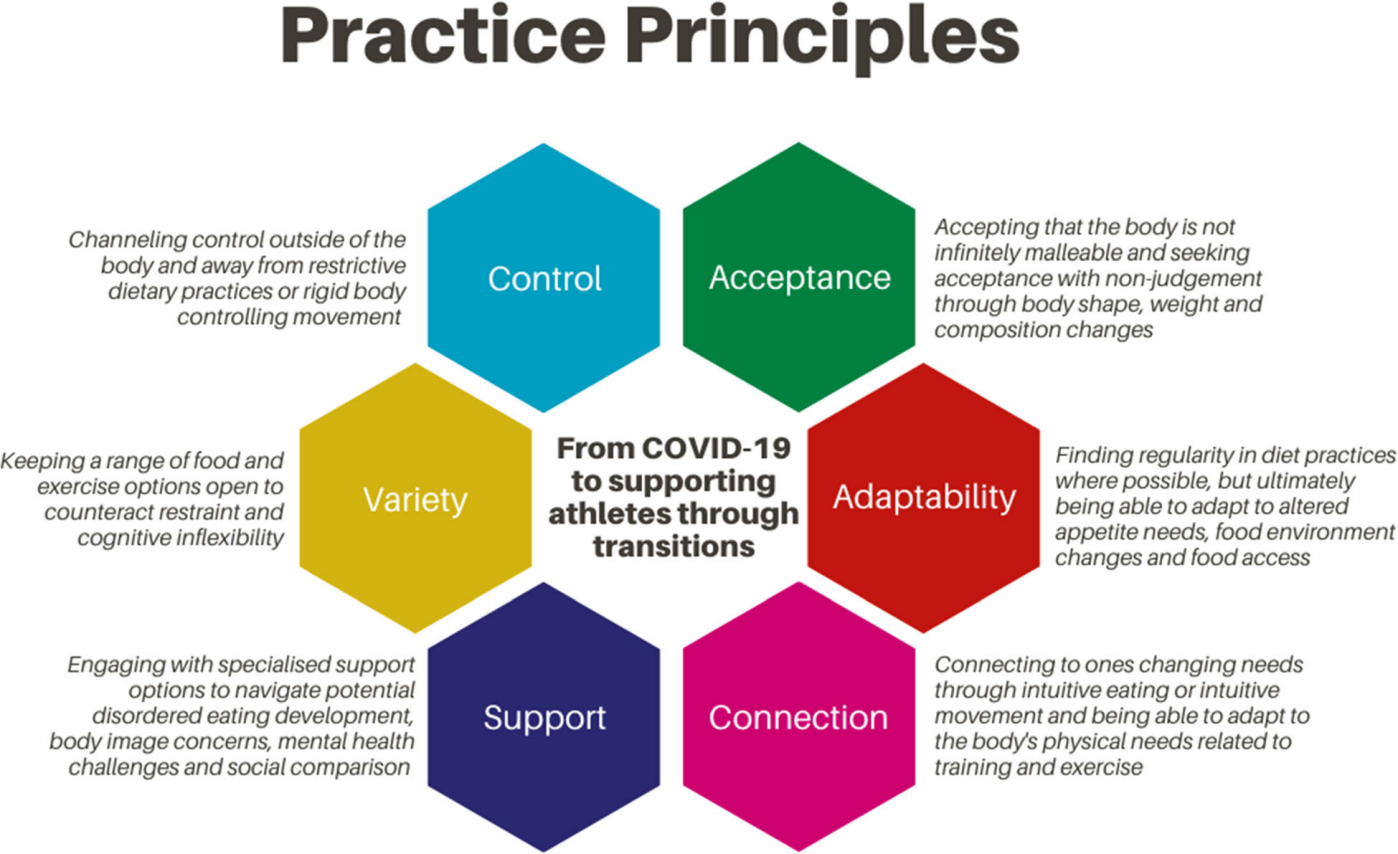
Practice Principles to Guide Individuals Engaging with Current and Former Athletes through Times of Transition Pertaining to the Food-body Relationship

Six key areas (Fig. 3) have been developed from the study findings as practice principles for those engaging with current and former athletes to assist in these transitions. These principles take into consideration DE prevention and have utilised the adaptive findings that participants described when their food-body relationship improved over this transition.

### Limitations

This study was conducted in the early stages of the international COVID-19 response and was designed to capture how athlete’s past and present relate to food and their bodies in transitions. The study population was limited by the male participant numbers (*n* = 29) and the subsequent variability of their EAT-26 reliability (0.45–0.88). To date there is no ED tool that has been developed with current and former male athletes at the forefront and as such, measurements are limited by their capacity to capture the phenomenon in such population groups. Further limitations include subjectivity surrounding the self-report questions and the open interpretations of the qualitative questions. The qualitative interpretations of “body image” and were unidimensional and primarily related to physical appearance despite the quality and breadth of information provided by participants. Further semi-structured interviews or focus groups would have been useful to capture nuance and tease out the complex and multidimensional experience of athletes’ relationship to their bodies.

Self-report questions require a level of psychological insight to be able to accurately capture an individual’s state. The findings of this study may be limited by the insight the participants had into what body image was and/or their personal interpretation of their food relationship. Individuals who come from sporting populations that normalise DE may also find that their insight into their condition is compromised based on their relative comparisons to their peers [72–74]. The prevalence of EDs in endurance, aesthetic, leanness-emphasising and weight class sports are said to have consistently higher rates of EDs compared to other sporting categories [15, 32, 55, 75]. This sample was derived from a population where 42.6% (*n* = 87) identified an endurance athlete and may be contributing to higher levels of EDs as a representative sample of mixed sports.

These results are by no means generalisable for all current and former athletes experiencing changes relevant to the COVID-19 pandemic. Every country, sport and individual has vastly different socioeconomic and geopolitical circumstances. For instance, endurance runners have largely been able to train unaffected in many countries, whereas swimmers or team sport athletes have faced significant barriers to train or perform their sport at all with the closure of pools and facilities [76]. For other individuals COVID-19 has meant the end of their sporting career with widespread collegiate team cuts seen in the USA [39, 40]. Some athletes have been able to find light in the darkness, develop resilience, new meanings and thrive in this time, whereas other athletes have been battling new challenges that have surfaced; particularly related to mental health, isolation and fitness deconditioning [41, 77, 78].

## Conclusion

A large number of current and former athletes have experienced worsened body image and disordered eating as a result of the acute COVID-19 pandemic response. This study highlights the importance of prevention efforts in sporting sectors to allocate funding and specialised support towards reducing body related anxiety, paradoxical food control, and maladaptive exercise management in times of transitions. We encourage future research to investigate the longitudinal and long-term effects of this negative transition on these at risk populations for ED development; both current and former athletes.

## Supplementary Information

**Additional file 1.**

## Data Availability

Interview data is available upon request in line with the Swinburne University institutional ethical approval SHR: 2019/113.

## Abbreviations

CMM: Convergent Mixed Methods
COVID-19: Severe Acute Respiratory Syndrome Coronavirus 2
DE: Disordered Eating
EAT-26: Eating Attitudes Test-26
ED: Eating Disorder
SARS-CoV-2: Severe Acute Respiratory Syndrome Coronavirus 2
USA: United States of America
