# Supplementary material for: Disordered eating & body image of current and former athletes in a pandemic; a convergent mixed methods study - What can we learn from COVID-19 to support athletes through transitions?
Source: J Eat Disord. 2021 Jun 24;9:73. doi: 10.1186/s40337-021-00427-3 (PMC8223527; doi:10.1186/s40337-021-00427-3)
Supplement: Supplementary file 1 — Additional file 1. [file 40337_2021_427_MOESM1_ESM.docx]

Supplementary Material

Qualitative Results Explaining improved or maintained body image or relationship with food

**Improved or Maintained Body Image**

For those who reported their body image was about the same, it was not uncommon to describe a paradox where both positive and negative aspects of body image were described. A current athlete explored the duality of feeling both negative and positive about their body, *“[I’m] feeling less fit but also being more patient and understanding with myself.”* Another current athlete described this paradox through their body image being conflated with their body composition, *“I’ve lost weight but some [of] it has been muscle, so I feel neutral.”* This paradox was further described by a former athlete, *“It’s been a constant struggle and I’ve still be able to exercise and eat healthy.”* For others, they found that their body image had not changed due to the previous work they have overcome in improving their relationship with their body. A former athlete described how actively working on their body image in athletic transitions has provided resilience in these difficult times, “*I’ve done extensive body image and food relationship work since retiring from sport. These unprecedented circumstances have proven that the work I have done has proven beneficial. I don’t feel fear for my body changing, although I know had this been a couple year ago, it would have consumed my thoughts.”*

A current athlete experienced improved body image through gratitude and appreciation for what their body is capable of since being injured, *“[I’ve] been able to train consistently without distractions and injuries again. And therefore appreciate what my body is doing for me and allowing me to train.”* A former athlete felt their body image had improved despite exercising less as they experienced gratitude for their health and less social comparison, *“I’m not as active working from home, however, I am grateful for my body and how I have been able to stay healthy during this time. I am not able to physically compare myself to people every day which has helped me too surprisingly.”* For others, being removed from their sporting cultures or social groups meant they experienced less objectification and body shaming, *“[I’m] not so preoccupied with looks because I'm by myself and nobody can see my body to judge it.”* This was experienced by both current and former athletes, *“…I don’t have to see people in public and worry about how they perceive me.”*

**Improved or Maintained Relationship with Food**

For participants who experienced a maintained relationship with food, it was not always a positive maintenance. A current athlete described how reduced access to their supermarket had provided temporary relief to symptoms aligned with binge eating disorder despite the underlying psychological features remaining unaddressed, *“Limiting grocery shopping to once or twice a week prevents me from going to the supermarket late in the evening if a binge urge kicks in, I make sure to only shop when I’m in a healthy food mindset so I don't buy anything I would normally binge on, therefore there isn't any of my go to binge foods in the house even if the urge does arrive.”* A former athlete described the battle they continue to face with their relationship with food, *“I still think about food and I still monitor my body and I can tell I am not exercising but I also don’t give a s**t. It feels like I will NEVER get to a good place with food so why bother?”* A current athlete further described the paradox of both positive and negative aspects of their relationship with food coexisting, *“I would say I go through phases of eating really well then have a bad day where I over eat.”* For others, it was a time to reflect on how far they had come in healing their relationship with food and were grateful for its consistency through this transient time, *“I have worked extremely hard to repair my relationship with food, and I am seeing how steadfast that work is proving to be. I feel free and flexible with my nutrition which is a very good thing considering grocery stores have empty aisles and foods I usually buy are not always available. Thankfully, I feel confident to make other food decisions that will still nourish my body well without guilt or fear.”*

An athlete described how the pandemic had afforded them more time to learn about nutrition and have gratitude for their food, *“I have spent more time cooking meals from scratch. Finding meals that are healthy and learning about the nutritional benefits.”* This was further explored in a participant’s appreciation for the resources they were able to access*, “I am grateful that we have resources in Australia to be able to make nutritious meals in this situation.”* A former athlete described using this time for growth and learning to reconnect with their body cues through intuitive eating, *“I’m trying to use this time to ditch dieting mentality and try intuitive eating. Earlier during lockdown I was very hard on myself but I’m easing up.”* A change in living environment was a positive change for a former athlete who found their parents role modelled a more balanced diet, *“Living with parents recently, who eat a more balanced diet than I do.”*
